# Structural and Electromechanical Insights into Thermoplastic Polyurethane/3D Hybrid Carbon Nanocomposites for Strain Sensor Applications

**DOI:** 10.1021/acsomega.5c08617

**Published:** 2025-12-26

**Authors:** Vaishnav B, Benedikt Sochor, Ajay Gupta, Sarathlal Koyiloth Vayalil

**Affiliations:** † Department of Physics, 28332 Applied Science Cluster UPES, Dehradun 248007 India; ‡ Deutsches Elektronen-Synchrotron DESY, Notkestrasse 85, Hamburg 22607, Germany; § Advanced Light Source, Lawrence Berkeley National Laboratory, 6 Cyclotron Rd, Berkeley, California 94720, United States

## Abstract

Incorporation of carbon allotropes of different dimensions within elastomeric matrices has been established as an effective strategy to fabricate functional conductive polymer nanocomposites (PNCs). In this work, higher-dimensional 3D hybrid carbon nanofillers, comprising synergistically integrated multiwalled carbon nanotubes immobilized onto few-layer graphene, were incorporated into the thermoplastic polyurethane (TPU) matrix to demonstrate their effectiveness as strain sensors. The conductive films were fabricated through a simple solution casting technique, in which the mechanical, electrical, and strain-sensing characteristics were studied in view of filler distribution, structural confinement, and interfacial interactions. Analyses using wide-angle X-ray scattering, Raman spectroscopy, and tensile testing revealed a higher degree of filler reinforcement within the TPU moieties, indicating pronounced interfacial interactions. Further, the tensile modulus increased significantly with filler loading above its percolation threshold (363% for 20 wt % loading). The structural features of dispersed filler aggregates were explored through an iterative model fitting of the ultra-small-angle X-ray scattering (USAXS) data, along with scanning electron microscopy (SEM). As a strain sensor, the films displayed a superior working-strain Gauge Factor (GF = 123, up to 8%), with exceptional stability under both unidirectional and cyclic strain. The findings provide a fundamental understanding while validating the potential of hybrid carbonaceous fillers for the fabrication of PNCs with futuristic applications.

## Graphical abstract

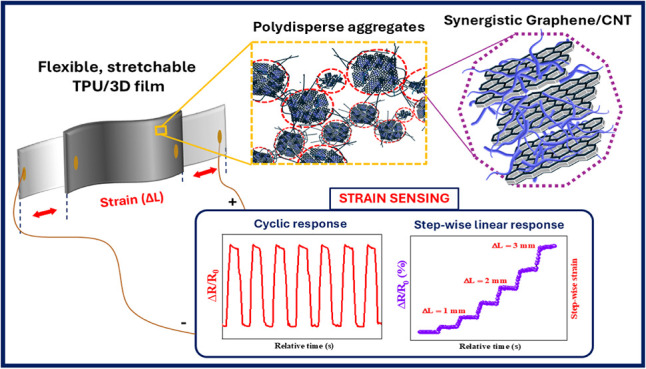

## Introduction

Flexible, conductive elastomers are integral components in various applications, including wearable electronics,^1–3^ health monitoring,^4–6^ smart textiles,^6–8^ etc. The increasing demand for smart, flexible materials has driven extensive research toward understanding, exploration, and engineering of novel elastomeric materials. The innate properties of elastomers, such as flexibility, softness, easier processability, and tunability, have been exploited to yield new materials with enhanced functionalities. The resulting properties of the elastomer are decided by the nature of nanofillers being incorporated; moreover, the concentration, distribution, and site-specific structure formation (aggregation) of nanofillers crucially decide the overall properties of polymer nanocomposites (PNCs).

Numerous studies on carbon-based PNCs have revealed that carbon nanoparticles efficiently enhance the electrical conductivity, as well as the mechanical and thermal stability of elastomers.^9–11^ Allotropes such as carbon black (CB), carbon nanotube (CNT), and graphene, possessing exceptional thermal and electrical conductivities (∼10^3^– 10^5^ Sm^–1^), high aspect ratios (1D and 2D nanoparticles), and huge intrinsic moduli (in the case of graphene), are well-suited to meet these necessities. In the context of strain-sensing behavior, the sensitivity (quantified as Gauge Factor (GF)), large working strain, and good cyclic stability were observed for CNTs. For example, Wang et al. fabricated a CNT/polydimethylsiloxane (PDMS) sensor to obtain a highly stretchable, sensitive strain sensor with tunable sensitivity for human motion detection.^12^ Park et al. introduced MWCNTs in polyethylene oxide (PEO) and reported superior sensing behavior compared to metal alloy strain gauges.^13^ However, the moderate dispersion and limited extent of linear strain sensing serve as constraints. The infusion of graphene, on the other hand, has elevated the conductance with increased linearity in sensing. Earlier, Zeng et al. prepared a very light reduced graphene oxide (rGO)/PDMS composite that resulted in a wide linear sensing range (up to 110%) and achieved a GF of 7.2.^14^ Boland et al. have shown an ultrawide working strain of 800%, with a GF of 35, demonstrating good cyclic stability in highly elastic natural rubber/graphene composites.^15^ Nonetheless, the limiting durability due to cracking effects and the relatively lower GF than CNTs is observed in the case of graphene incorporation. Therefore, a synergistic approach involving the integration of CNTs with graphene was employed to harness the effects of both 1D and 2D fillers. In the recent past, studies have shown the applicability of 3D-filled PNCs for different applications. For example, Wang et al. have shown that the dispersion of graphene oxide (GO)/functionalized CNT (f-CNT) heterostructure hybrids within the PDMS matrix displays a 20-fold increase in thermal stability.^10^ Roy et al. have reported an increase in the storage modulus of thermoplastic polyurethane (TPU) by 206%, along with a significant increase in thermal stability by introducing multiwalled CNT (MWCNT)/rGO hybrids.^11^ Given its functionality, a detailed understanding of the structure–property relationship is essential.

In a highly polar matrix such as TPU, the carbon fillers tend to form hierarchically distributed structures. Such structures tend to form conductive network pathways, enhancing the transport characteristics of the composites. Hierarchical aggregate distribution, in other words, their polydispersity, is governed by the balance between the dual interfacial interactions^16^ (i.e., interfiller and matrix–filler interactions), which stresses the importance of understanding filler morphologies in the light of interfacial interactions. In our previous studies, we have investigated such hierarchically distributed CNTs and wrinkled graphitic nanofiller structures through the X-ray scattering technique, considering the effect of matrix polarity.^17,18^ The elucidation of such embedded morphologies within matrices requires a nondestructive yet transmissive probing technique, which primarily is the small-angle X-ray scattering (SAXS).^19,20^ The minimum and maximum length scales that could be probed by this technique rely on the maximum and minimum resolvable scattering vector (*q*) by the measurement (for SAXS ∼ 1 < *D* < 100 nm), which are decided by the technical limitations. To elucidate larger structures, as in our case, is the aggregates (a few nanoparticle clusters), the limitation is overcome by the ultra-small-angle X-ray scattering (USAXS) technique (resolves ∼ a few hundred nm). This inverse space imaging technique has its own advantages over real-space imaging, where it has been shown to elucidate structure on multiple length scales.^20,21^ Extensive structural elucidation in CB,^22,23^ CNT,^24–26^ and graphene^27,28^ based PNCs was done using this technique, thereby establishing its applicability.

In this work, we investigate the enhancement effects in TPU as a result of 3D nanofiller dispersion, considering the filler distribution, structural confinement, and interfacial interactions. The mechanical, electrical, and electromechanical or strain-sensing characteristics of the films have been comprehensively analyzed. Herein, we have utilized the synergistic few-layer graphene (FLG) with f-MWCNTs as our 3D hybrid nanofillers, which are dispersed in TPU via a solvent-mediated technique to yield thin, flexible, conductive films. The filler fractions are chosen in such a way as to examine the composites’ behavior at, above, and below their percolation threshold. The USAXS technique has been employed to explore aggregate morphologies of 3Ds, which are then complemented by other *ex situ* characterizations. A further deliberate structural elucidation is performed through an iterative model fitting of the USAXS data. The electromechanical performance has been systematically investigated to explicate its applicability as a strain sensor, which resulted in both heightened and stable sensitivity. This study provides a comprehensive understanding of the structure–property relationship in 3D carbon-based elastomeric composites.

## Materials

A commercially available aliphatic polyether-based TPU, known as Texin SUN-3006, is an extrusion-grade material with a hardness rating of 90 Shore A, a specific gravity of 1.08 g/cm^3^, and a characteristic molecular weight of approximately *M*_n_ = 79 000, *M*_w_ = 2 11 000, and melt flow index (MFI) of 5.1 g/10 min at 200 °C with a load of 2.16 kg. TPU was sourced from Covestro, India. The organic solvent *N*,*N*-dimethylformamide (anhydrous, 99.8%) (DMF) was procured from Sigma-Aldrich, while the other required chemicals were locally procured.

### Preparation of TPU/3D Composites

The elastomeric TPU composites were prepared via solvent-mediated nanofiller dispersion through the Doctor’s blade method, as shown in Scheme 1. Among the different casting techniques, a geometrically stable, well-defined thin film with uniform thickness was reported to be obtainable through this method.^29^ In a typical solvent-mediated technique, a solvent reduces the polymer melt’s viscosity, improving the mobility of chains. This results from the unfolding of polymer chains, improving their dynamics, which enhances the phase intrusions within them. The hybrid 3D carbonaceous nanofillers used in this work were synthesized in the laboratory by immobilizing multiwalled carbon nanotubes (MWCNT–COOH) onto edge-functionalized few-layer graphene (FLG-OH), whose details are mentioned elsewhere.^30^ In a typical process, functionalized MWCNTs were introduced into the organic solution (0.1 wt % in NMP) that contained graphite, which had undergone prolonged exfoliation overnight to result in FLG. The introduced MWCNTs were allowed to undergo interfacial integration under sonication, after which settling and centrifugation were carried out to obtain a stable dispersion with CNTs firmly embedded into graphene flakes. For the synthesis, a measured quantity of 3D nanofillers was subjected to extended sonication in an organic solvent (DMF) to produce a well-dispersed suspension. For the study, the nanofiller concentrations were chosen to be 10%, 15%, and 20% of weight (wt %) of TPU (with ∼12 wt % being the characteristic percolation threshold). On the other hand, a calculated amount of TPU (at 2 mg/mL) was dissolved in the same solvent at 90 °C to produce a homogeneous solution. This optimized temperature was necessary to facilitate efficient gelation kinetics in the phase-segregated TPU. The sonicated filler suspension was introduced into the TPU solution and vigorously stirred until homogeneity. The stirring continued under heat to evaporate the solvent to eventually achieve a viscous solution, which was then poured onto a preheated aluminum mold of dimensions 30 × 10 × 1 mm. Over the casting surface, a Doctor blade was swept to remove the excess gel to obtain a uniform film. Further, multiple vacuum degassing steps were done to remove trapped air bubbles during the course of solvent evaporation. A thin, free-standing, phase-segregated film was then obtained through a thermoreversible gelation. The prepared samples are denoted by the names PUD10, PUD15, and PUD20, corresponding to 10, 15, and 20 wt % of 3D filler-loaded TPU, respectively.

**Scheme 1:**
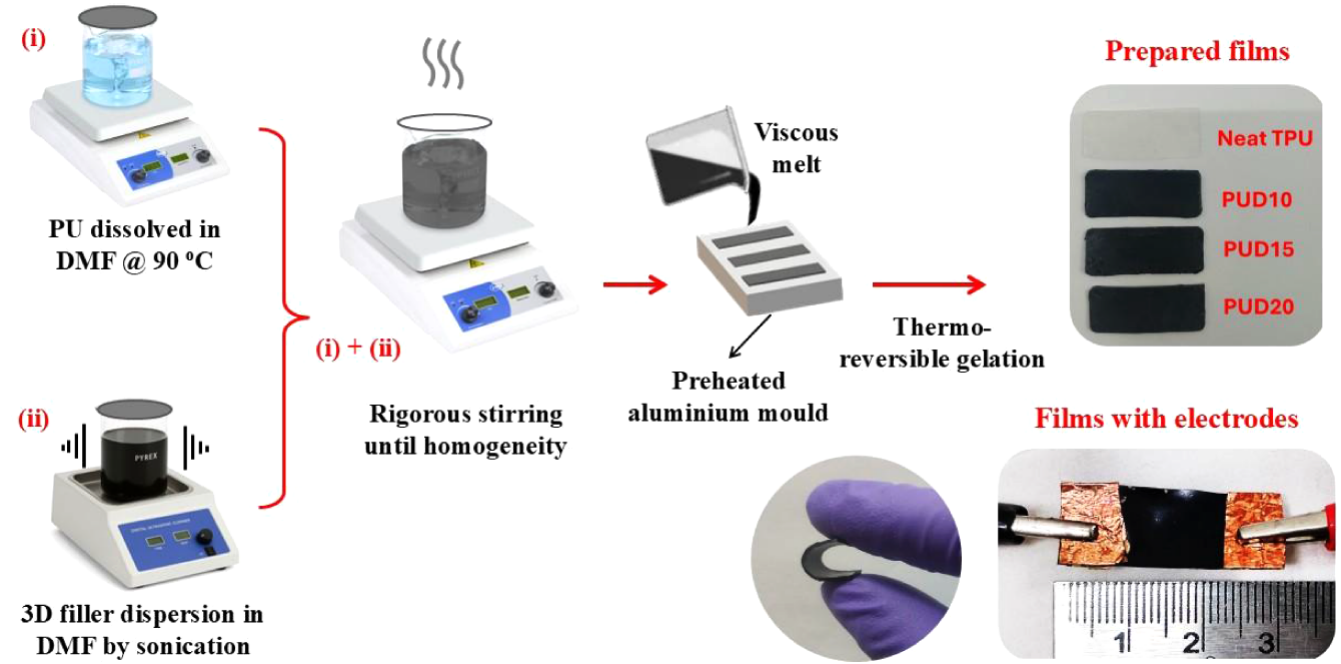
A Schematic Illustration of the Composite Preparation Process[Fn sch1-fn1]

a
Representative images of the prepared films with electrodes attached for conductivity measurements are presented.

### Characterization Techniques

The analysis and characterization of the prepared films were performed through different techniques as follows: Wide-angle X-ray scattering (WAXD) studies were conducted to observe the crystalline and phase composition in neat as well as 3D-incorporated PU composites using a lab source, Cu Kα radiation with a Panalytical Empyrean diffractometer. The data were collected for a range of 2θ between 10° and 65° at a step size of 0.05°. The micromorphological imaging was done using an Axia Chemi scanning electron microscope by Thermo Fisher Scientific, USA, at an accelerating voltage of 30 kV. The surface features of the as-prepared samples before and after the introduction of cyclic deformation were imaged. To observe the internal conformation of filler aggregates, cross-sectional scanning electron microscopy (SEM) was performed on finely sectioned thin strips. The surface of the strips was precisely polished to avoid the topmost layer and to expose internal conformities. The Raman spectra for neat as well as composite samples were recorded using a Raman Spectrophotometer (RIMS-U-DC) using a 532 nm laser source, at a power of around 1 mW. The spectra were recorded between 500 cm^–1^ and 3000 cm^–1^. The temperature-dependent storage modulus of the films was measured by using the device, DMA 850, by TA Instruments. Multiple measurements were carried out on neat as well as composite TPU films under tensile mode in a dual-screw clamping geometry. The temperature range was between 25 and 100 °C, at a heating rate of 3 °C/min. The modulus was measured at a constant frequency of 1 Hz, at an amplitude of 20 μm, with a preload force of 10^–2^ N.

The strain-sensing behavior of the prepared conductive composites was thoroughly investigated using an in-house-developed stretching device. The respective programmable device is capable of introducing controlled, stepwise cyclic strain at the desired strain rates. In this study, stepwise stretching, as well as repetitive stretch–relaxation cycles at different strain rates, with intermediate delays in each step, was imparted on the samples. The conductivity measurements were performed using a Keithley source-cum-multimeter (model 2450), which recorded varying current as a function of strain under a constant applied voltage of 20 V. Conductive leads for the measurements were created using a thin copper strip adhered to either end of the sample surface using silver paste.

The variation of electrical resistance as a function of applied strain was quantified using the conventional parameter Δ*R*/*R*_0_ (where Δ*R = R – R*_0_, with *R* being the instantaneous resistance under strain, and *R*_0_ is the resistance without strain). To examine the sensitivity of composite films, the dimensionless parameter GF is calculated using the following equation:

1 GF=ΔRR0ε

Where ε denotes the imparted strain given as Δ*L*/*L*_0_ (Δ*L* and *L*_0_ being the change in length and the original length, respectively). GF apparently denotes the net variation in resistance upon unit strain, signifying the sensitivity of a sensing element.

### Ultra-Small-Angle X-ray Scattering (USAXS)

To investigate the structural configurations of 3D fillers within TPU, USAXS measurements were carried out at P03 beamline of PETRA III, DESY, Germany.^31^ The transmission USAXS experiments were conducted at an X-ray energy of 11.8 keV, corresponding to a wavelength of 1.044 Å, with a sample-to-detector distance (SD) of 9550 ± 1 mm. The incident X-ray beam, with a size of 23 × 27 μm^2^ (horizontal × vertical), was scanned through 500 μm-thick samples. The scattered X-rays from the sample were recorded by using a PILATUS 2M detector (Dectris, Baden, Switzerland) with a pixel size of 172 μm. SD was chosen to be significantly large to resolve lower values of the scattering vector (*q*) that emphasize relatively large structures. This consideration was made because the composites were expected to possess hierarchically agglomerated 3D filler within them.^16^ The intensity varies with respect to the scattering vector, *q,* which is given as:

2 $$q=\frac{4\pi }{\lambda }\sin\left(\theta \right)$$

Where λ is the wavelength of the X-ray and θ is half of the scattering angle.

USAXS images recorded from a 2D detector were processed using the DPDAK software suite^32^ to produce 1D scattering plots. These intensity (*I*) vs *q* plots were obtained through azimuthal integration of pixel-wise intensity from the beam center, which is referred to as cake integration. The background subtraction and masking were done by following the procedures explained in the literature.^33^

### Scattering Model

Elucidation of structural information from a standard scattering pattern can be performed through conventional model-dependent as well as model-independent analysis. Various scattering models incorporating the form and structure factors, along with the possibility of combining more than one structure factor, enable critical investigation of the scatterers. In this study, a simplified and more generalized, shape-independent *Guinier–Porod model* has been utilized to fit and analyze the scattering data. The Guinier–Porod model has been shown to determine various shapes, including symmetric, asymmetric, as well as intermediate structures. The generalized function is given as:

3 $$I\left(q\right)=\{\begin{array}{cc} \frac{G}{q^{s}}\exp\left(-\frac{q^{2}R_{g}^{2}}{3-s}\right) & ,q\leq q_{1}\\ \frac{D}{q^{m}} & ,q\geq q_{1} \end{array}$$

Where *G* and *D* are Guinier and Porod scaling parameters, *R*_g_ is the radius of gyration, *q* is the scattering vector, *q*_1_ is the crossover between Guinier and Porod regimes, *s* is a shape-dependent dimensionality parameter, and *m* is the Porod exponent. A more elaborate theoretical discussion on this model can be found elsewhere.^34^

For fitting the respective function to the experimentally obtained USAXS data, SasView software has been used. SasView is an open-source software package comprising a variety of scattering models for fitting small-angle scattering data. This inverse-space fitting tool undergoes iterative least-squares optimization on *I­(q)* data sets for the selected model. Additionally, user-defined constraints can be assigned to ensure a physically reasonable estimate of the parameters. During fitting, the DREAM algorithm was employed, and the fitting was carried out for the entire *q* range. The detailed aspects of the fitting process are discussed in the following section.

## Results and Discussion

WAXD studies of the pristine TPU film, 3D fillers, and their composites depicting their crystalline and phase characteristics are shown in Figure 1a. A broad peak ranging between 13° to 30° centered at a 2θ of 20.03° for pure TPU arises from the well-known planar reflection (110), depicting the typical interchain distance of 4.43 Å. In addition to the presence of soft and hard segments, which are of short-range order, the broadened peak suggests the presence of amorphous TPU moieties.^35^ The intense (002) planar reflection from pristine 3D fillers at 2θ = 26.61° is a characteristic of the interlayer spacing corresponding to 0.335 nm.^36,37^ The two disrupted peaks at 42.42° and 44.58° are attributed to (100) and (101) graphitic planes. Together with (002), relatively feeble reflections from (004) planes at 2θ = 54.71° are indicative of the lamellar graphitic planes of FLG.^36^ It is to be noted that the (002) peak positioned at 26.61° rather than 26.75° of graphite further suggests the presence of exfoliated C = C layers to result in few layers. For the case of nanocomposites, a conventional increase in the intensity of the (002) and (004) reflections corresponding to graphitic planes is observed with incremental filler loading. Contrarily, the intensity of the (110) peak corresponding to TPU shows a decremental behavior, which could be due to the decreased crystalline phase of TPU upon filler intrusion within the polymer chains.

**Figure 1:**
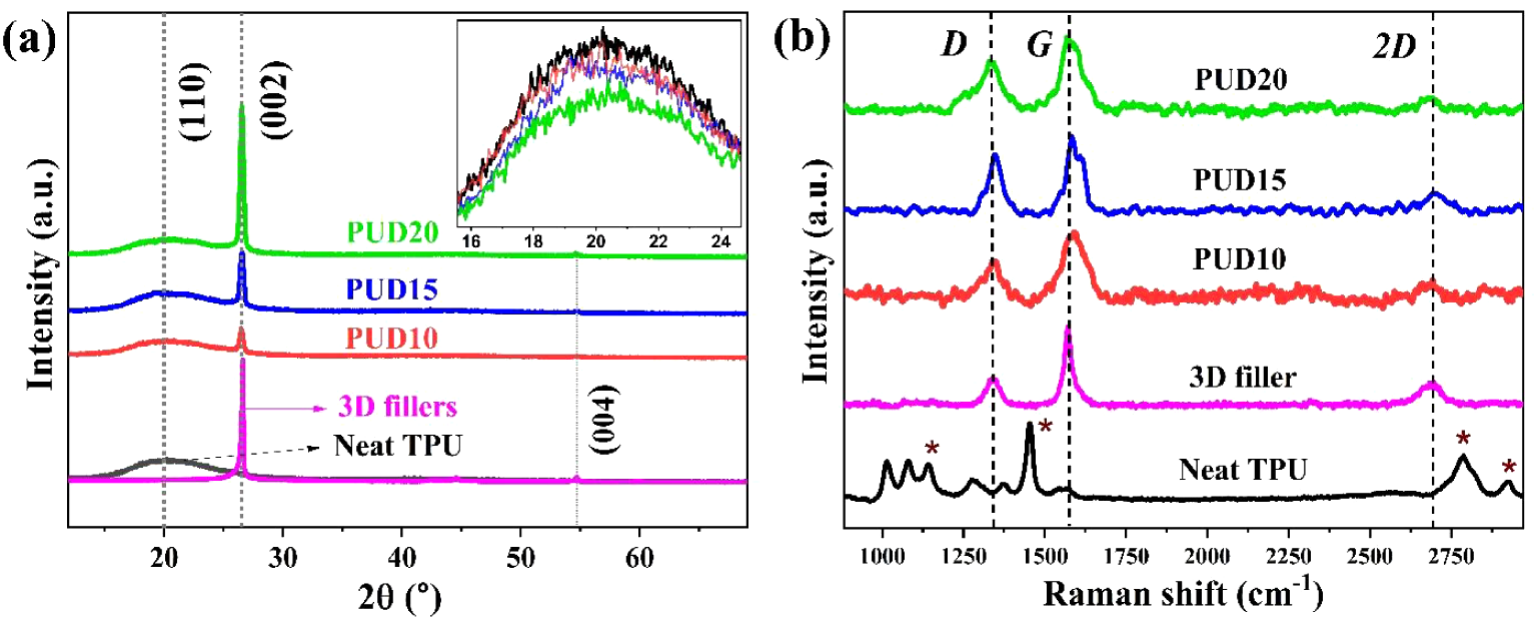
(a) WAXD patterns of neat TPU, 3D, and its composites shown with offsets for comparison. The inset in (a) shows the decrement of TPU’s (110) peak intensity with filler concentration. (b) Raman spectrum of the samples with the characteristic peaks of neat TPU: D and G and 2D peaks of the 3D fillers being highlighted.

The Raman spectra of TPU, 3D, and their composites are shown in Figure 1b. From the spectra of 3D fillers, we evidence the characteristic D, G and 2D bands at 1340 cm^–1^, 1570 cm^–1^, and 2689 cm^–1,^ respectively. The presence of a 2D band is indicative of FLG, arising due to the second-order overtone of in-plane transverse optical phonons from sp^2^ hybridized carbon atoms, which is sensitive to interlayer interactions and stacking.^38^ The G and D bands, respectively, are due to the E_2g_ phonon mode that represents in-plane vibrational symmetry, and lattice disorders in sp^2^ hybridized carbon structures. The spectra of neat TPU consisted of the prominent peaks at 1445 cm^–1^, corresponding to CH_2_ bending; two peaks at 2922 cm^–1^ and 2785 cm^–1^, which are associated with CH_2_ stretching; a peak at 1080 cm^–1^ corresponding to C–O–C stretching.^39^ In the case of composites, the characteristic D, G and 2D bands persisted, while the intensity of the 2D band diminished. Similarly, the peaks corresponding to CH_2_ bending (between 2700 and 2930 cm^–1^), C–O–C stretching (at 1080 cm^–1^) also exhibited a significant decrease in intensity. This is due to the reduced chain mobility in TPU moieties as a result of incremental 3D reinforcement.

### Structural Conformation of 3D Nanofillers

The macroscopic characteristics of elastomeric nanocomposites are greatly influenced by the nature of the distribution and degree of agglomeration of nanofillers within polymer matrices. Henceforth, to explicate the structural conformation of 3D nanofillers within TPU, we have performed SEM as well as USAXS studies on the samples. In addition to the as-prepared nanocomposites, we have imaged the same films after the introduction of cyclic strain to understand the stress effects on their morphological features. From Figure 2a to c, the comparative SEM micrographs elucidating the surface characteristics of as-prepared and strain-introduced PUD15 films are shown. It has been observed that, upon the introduction of mechanical strain (20% as shown in Figure 2b), the surface of the films gets wrinkled due to the strain-localization effect.^40^ When the films were subjected to multiple stretch–contraction cycles at larger strain values (>30%), we observed multiple strain-induced fracture sites, a few of which are shown in Figure 2c. These surface deformations usually occur for strains above their elastic limit. Such disruptions result in irreversible sheet resistances during cyclic stretching.

**Figure 2:**
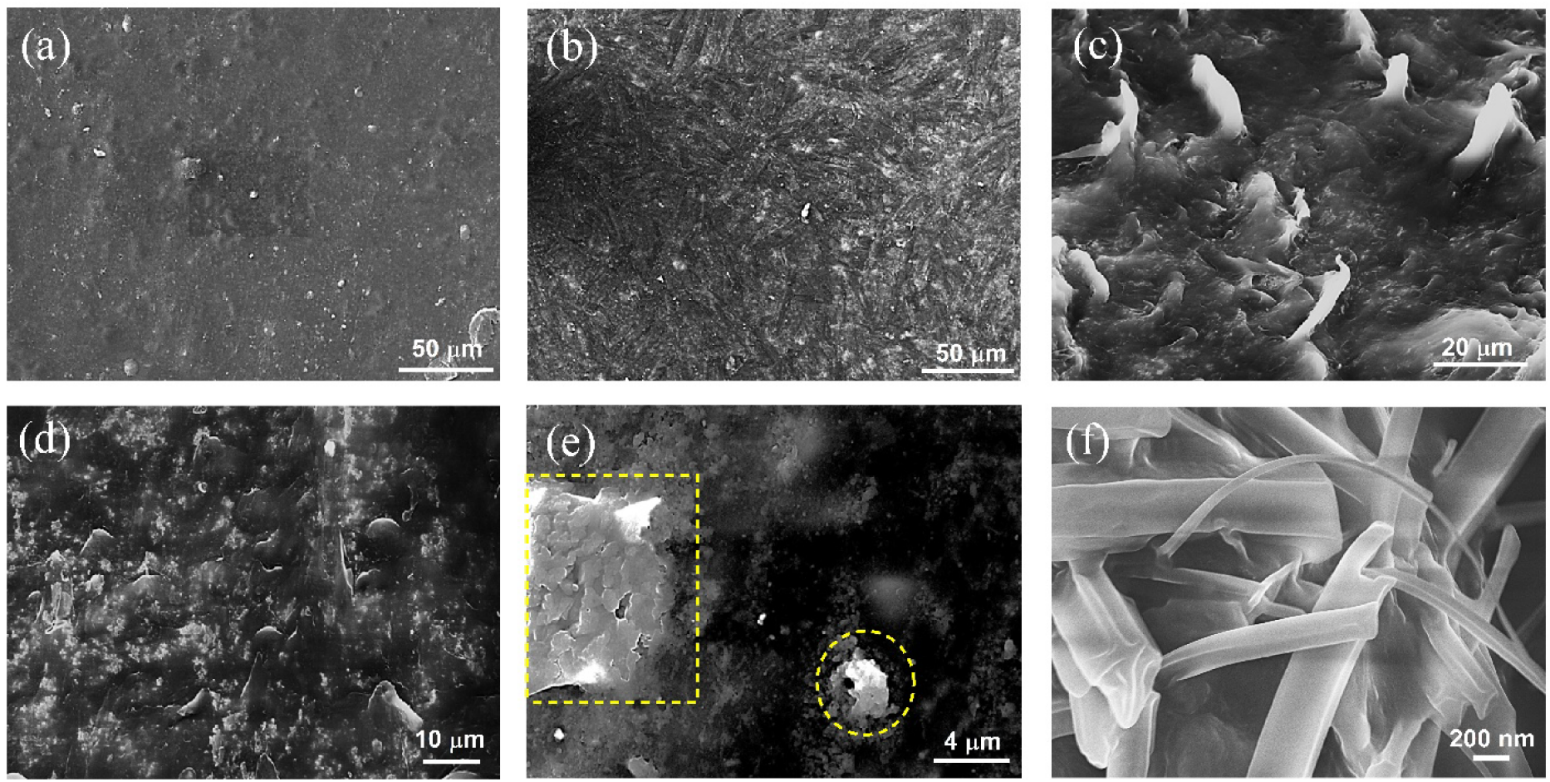
(a) and (b) represent the SEM micrographs showing the surface features of the as-prepared PUD15 film and its wrinkled surface after the introduction of 20% strain due to strain localization, respectively. (c) Shows strain-induced distortions at a larger strain (>30%). (d) and (e) are the cross-sectional images of the PUD20 sample, showing polydisperse aggregates and agglomerates, and (f) shows the synergistic 3D fillers within the TPU matrices.

Following the surface evaluation, cross-sectional SEM imaging was performed to explore 3D structures. It is worth mentioning that, from both cases, viz. SEM and USAXS, the hierarchical conformation of 3D filler structures in a polydisperse fashion within the matrices has been evidenced. This aspect has been widely discussed in carbon nanofiller systems, including our previous studies on CB and CNTs within polymer matrices.^17,18^ The representative 3D filler structures within PUD20 samples are shown in Figure 2d- f, indicating aggregate distribution, a few magnified agglomerates, and primary particles (a few particle clusters), respectively. In each case, no uniform or correlated size distribution is observed. In other terms, the distribution of nanofillers within TPU is polydisperse in nature. It is expected that such a distribution elevates the number of short contacts, favoring overall electron transport.

### Inferences from USAXS Model Fitting

1D scattering plots of the TPU/3D composites are shown in Figure 3. From the double logarithmic plots, one can observe that there are no characteristic peaks, which immediately infers the absence of strongly correlated or ordered structures in the system. To interpret the structural conformities of 3D fillers, a simplified Guinier–Porod model has been fitted to the obtained scattering data, which are shown in Figure 3. It is to be noted that the filler structures (including their agglomerates) are not confined to an explicitly definable geometry. Hence, a standard, shape-independent model that could account for the nonsymmetric structures is being considered for our case. One should note that the Guinier–Porod model does not account for indexing the polydispersity of scatterers. Hence, the degree of polydispersity is unquantifiable under the present consideration.

**Figure 3:**
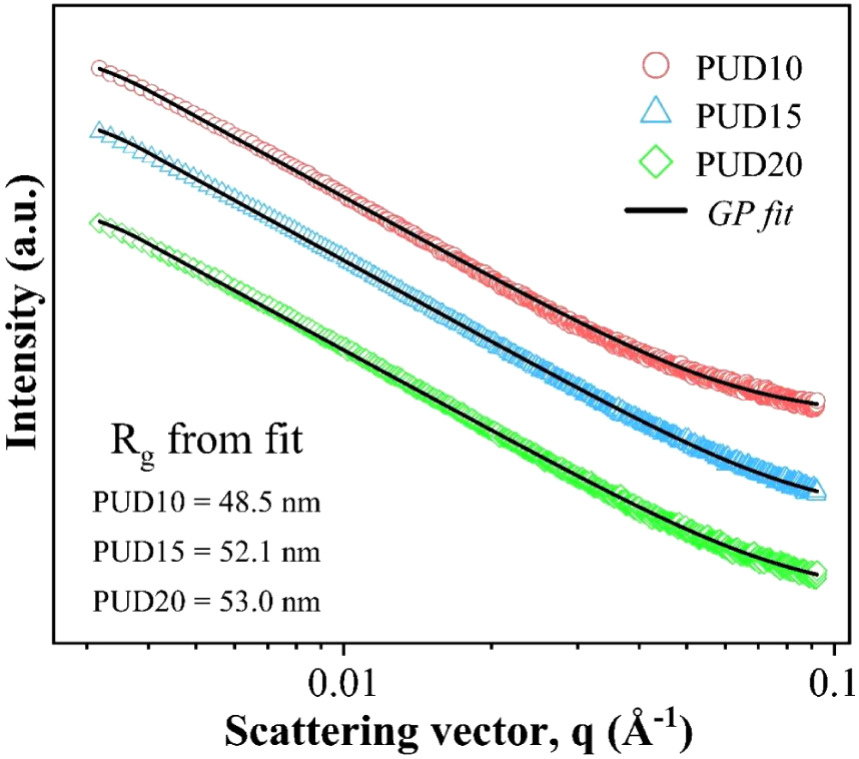
1D scattering curves of TPU/3D composites, along with the fitted data, are shown as a solid line. The incremental *R*_g_ obtained from the fitting is mentioned within the graph.

The structural parameters, viz. *R*_g_, *s*, and *m* that define size and shape have been obtained from the best fits. In the present case, aggregate conformations are the structures of interest, according to which the constraints are defined for parameters. The obtained values, along with fitting parameters, are shown in Table 1. The minimum and maximum values of *R*_g_ are chosen in such a way that they correspond to the extrema of probed *q* values (with 0.0318 nm^–1^ < *q* < 0.922 nm^–1^) guided by the equations: *D =* 2π/*q* and *R*_g_ *= R*(3/5)^0.5^ =(*S*/2) (3/5)^0.5^, where *S*/2 is the generic half-size^41^ given as:

4 $$\left(S/2\right)=R_{g}\sqrt{\frac{5-s}{3-s}}$$

**Table 1:** Results Obtained from the Guinier–Porod Fitting on the USAXS Data^a^

*	*s* (-)	*m* (-)	*R*_g_ (nm)	S/2 (nm)
PUD10	0.02 ± 0.08	2.94 ± 0.01	48.5 ± 1.8	62.6 ± 2.3
PUD15	0.09 ± 0.05	2.97 ± 0.03	52.2 ± 1.7	67.4 ± 2.2
PUD20	0.03 ± 0.04	2.95 ± 0.11	53.0 ± 2.1	68.4 ± 2.7

a The standard uncertainties in the obtained values of *R*_g_ are around 3%, which could be accounted for by lower error estimates and unresolved structural aspects. The lower error estimates could be attributed to the large number of data points being fitted, where a very minimal residue on each data point accumulates into a considerable value.

The equation corresponding to the radii of globular spheres was derived, since the preliminary fittings consistently yielded the value of *s* close to zero for all composites.

From the obtained values, information about the nature of scatterers could be inferred comprehensively. To start with, we observe that the value of the dimension-indicative parameter *s* is nearly zero for all the composites. From the theory of the Guinier–Porod model, the dimension of scatterers is defined through the relation (3 – *s*), which implies that the structures are cylindrical or lamellar when *s* is 1 or 2, respectively. Hence, *s* ≈ 0 approximates that the aggregate conformities (on a global average) are globular structures.^34^ Due to Van der Waals forces between the fillers in the vicinity, the aggregates inherently tend to confine symmetrically. Yet, the functionally rich TPU matrix tends to elicit its contribution to give rise to matrix–filler (or interfacial) interaction. It can be seen from SEM images that the clusters lacked symmetrical morphologies. Hence, the resultant structures are a result of the balance between the two interactions.

The Porod exponent, *m*, quantifies the nature of the interfaces and fractal characteristics of the scatterers. For instance, a Porod exponent nearly equal to 4 generally indicates smooth, compact interfaces. If the exponent lies between 2 and 3, it suggests scattering from mass fractals. Contrary to the previously reported value of *m* between 3 and 4 for lamellar graphitic structures with surface fractals,^42^ and even lesser (1< *m* < 2) for CNTs,^43^ the 3D structures display an intermediate behavior. The obtained values of the exponent (*m*) bridging between the mass and surface fractals depict the scattering from branched, irregular networks.^34^ Such branched structures of 3D aggregates could be accounted for by the formation of transport networks within the TPU/3D composites. Moreover, the fit has yielded *R*_g_ values of ∼48.5, 52.2, and 53.0 nm, corresponding to generic half-sizes of 62.6, 67.4, and 68.4 nm for PUD10, PUD15, and PUD20 samples, respectively. Since *R*_g_ quantifies the average size of scattering domains present in the composites, the obtained values indicate a consistently increasing aggregate size with filler concentration. It is understandable that the incremental filler loading results in an increased number of larger-sized clusters due to pronounced filler–filler interaction compared to matrix–filler interaction. From the electrical transport studies, we have observed that the percolation threshold is just above 10 wt %; concurrently, we see that there is a relatively significant increase in average aggregate size just above the percolation threshold—i.e., between 10 and 15 wt % in comparison to the increment observed between 15 and 20 wt %. Moreover, the subsequent addition of nanofillers may result in a relatively decreased rate of aggregation in matrices. This accounts for the fact that just before percolation, the fillers span the matrix and a feeble loading above percolation onsets the bridging of interfacial transition zones between isolated clusters and primary particles. This typical transition in morphology at percolation has been previously observed in carbon black aggregates.^44,45^ It is affirmative to observe the respective behavior in a dimensionally distinct 3D nanocomposite system as well, since this suggests that 3D fillers also obey similar percolative behavior as that of other carbon fillers in polymer matrices.

### Mechanical Characteristics

DMA analysis has been carried out to understand the interfacial interactions between the 3D filler and the TPU matrix. In our previous study,^17,18^ we observed the effect of one- and two-dimensional carbon fillers, viz. MWCNT and graphene addition in polar and nonpolar matrices. It has been observed that the intermolecular interactions between the moieties are critically affected by the temperature, which alters the polymer chain dynamics. It has also been reported that the carbon filler infusion within matrices not only affects the mechanical characteristics but also amends the critical temperature (such as glass transition) of composites.^46^ Here, we have studied the (tensile) modulus of the samples in a functional temperature range of typical strain sensors, which is between room temperature (RT ∼26 °C) and 100 °C.

From the plot shown in Figure 4, one can clearly see a consistent surge in the storage modulus of TPU with the addition of nanofillers. The room-temperature storage modulus of the samples has been observed to be ∼34.7, 54.2, 91.1, and 160.7 MPa for neat TPU, PUD10, PUD15, and PUD20 samples, respectively. Clearly, an increment of 56% in modulus with 10 wt % nanofiller addition has been observed, while a significant surge of 163% and 363% was observed for 15 and 20 wt % loading, respectively. This surge in modulus suggests the effect of 3D reinforcement within polymer moieties, resulting in a diverse mechanical behavior of the nanocomposite. TPU, being functionally rich, shows a higher degree of interfacial interaction with the 3D carbon interfaces. The intrusion of a 3D filler interrupts the dynamics of TPU polymer chains, resulting in an increased rigidity of composites. It is seen from the plot that the modulus decreases with an increase in temperature, which is attributed to increased chain mobility. The predominant matrix–filler interaction, hence, suppresses the emergence of active polymer chains—onset by an externally applied temperature.

**Figure 4:**
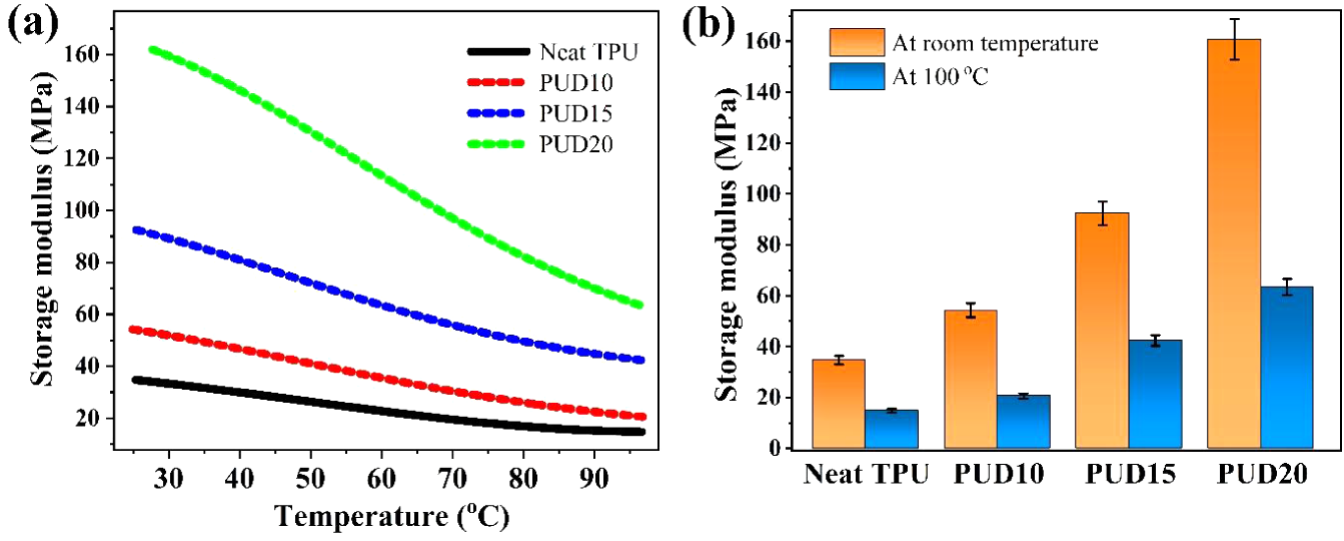
(a) The temperature vs storage modulus curves for neat and 3D-incorporated TPU at different loading fractions, and (b) compares the modulus at room temperature and 100 °C, indicating a systematic increase in modulus with filler loading.

From the degree of linearity in modulus versus temperature curves, one can understand the stability of filler networks within composites. A typical nonlinear fall with temperature is observed at higher filler loadings, which is attributed to the breakdown of filler networks within elastomers.^47^ Henceforth, we can infer from the data that the 3D networks are significantly stable with temperature within TPU matrices, which is beneficial for applications. To achieve enhanced electrical conductivity, the infusion of larger amounts of nanofillers may befit, but at the cost of reduced flexibility of the composites.

### Electromechanical Characteristics

The electromechanical studies, which depict strain-sensing capabilities of the prepared conductive films, have been systematically investigated under controlled unidirectional as well as cyclic strain. From the measurements, it was observed that the PUD10 composite does not show considerable electrical conductivity, which is because the filler loading is less than its electrical percolation threshold. On the other hand, an enhanced conductivity, with an excellent strain response, is observed for PUD15 and PUD20 composites. This shows that TPU/3D composites produced through the simple solution casting possess a percolation threshold between 10 and 15 wt %.

The obtained strain–conductivity results are listed in Figure 5. The stability of sensing has been tested by applying a uniform, stepwise strain up to 10% of its nominal length. An incremental strain in steps of 0.5 mm, at a strain rate of 0.5 mm/s, with a relaxation time of 2.5 s in each step, has been applied, and the respective electrical responses were recorded. The obtained results are shown in Figure 5a and c. Both films displayed an excellent response upon loading with appreciable stability in each step. It is clearly evident from the results that the relative resistance change (Δ*R/R*_0_) is significantly higher for the PUD20 composite compared to PUD15. This is attributed to the higher concentration of 3D in PUD20, which facilitates the formation of a greater number of conductive network pathways. Furthermore, while imparting strain, a proportionally greater number of network breakdowns occur, which is reflected in the form of decreased electrical conductivity.

**Figure 5:**
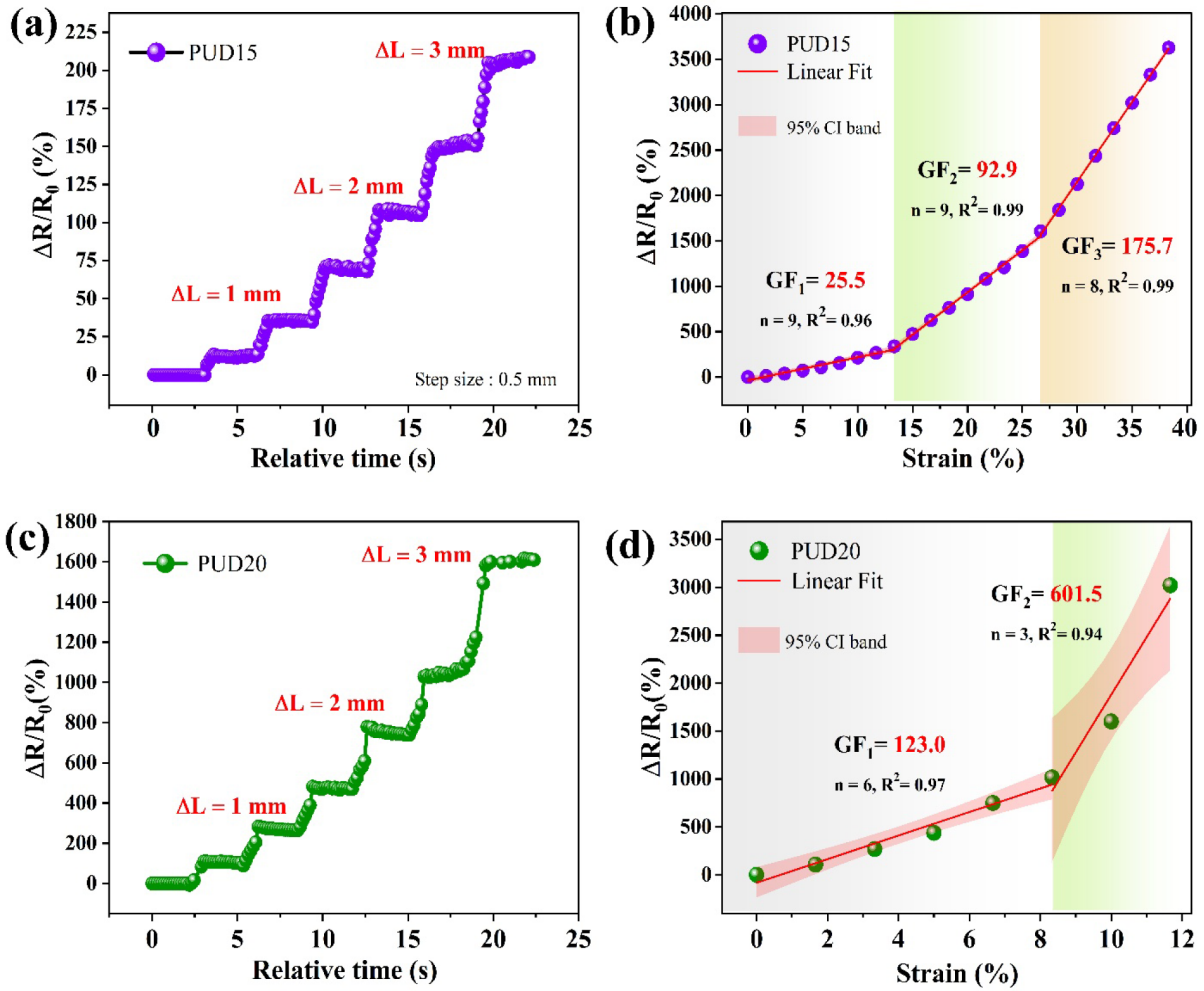
(a) and (c) show the strain-dependent resistance change in PUD15 and PUD20 films, respectively, under a constant stepwise strain, with a relaxation at each step to show their sensing stability. (b) and (d) illustrate strain vs Δ*R/R*_0_ plots of PUD15 and PUD20, respectively, which are fitted linearly to obtain GF in different stages of strain. The shaded regions in (b, d) bound the 95% confidence interval band of the performed linear fits, with *n* being the number of data points considered in each regime. The *R* ^2^ mentioned within the graphs indicates the coefficient of determination, depicting the goodness of fit.

In order to determine the extent of linearity in sensing, the Δ*R/R*_0_ with incremental strain up to elongation at the break has been recorded. The results are shown in Figure 5b and d. To determine the GF, a linear fit was applied to the corresponding data, as indicated by solid lines in the plots. A linear GF (denoted as GF_1_) of 25.5 has been observed for PUD15 composites, while PUD20 has resulted in a very high value of 123. This shows that the higher concentration provides greater sensitivity; however, the extent of linear sensing is limited. In the case of PUD20, the linear range is observed up to 8% strain, while it is 13% for PUD15. This limitation with higher concentration is due to the increased mechanical rigidity with loading, which has been evidenced from DMA studies. The small strain sensitivity, GF_1_, is predominantly due to progressively limited tunneling conductance as a result of increasing interfiller distance. GF_2_, as in the case of the PUD15 nanocomposite, corresponding to intermediate strains, is a net resultant effect between network breakdown and strain-induced reordering, while GF_2_ in PUD20 and GF_3_ in PUD15 show an abrupt increase in sensitivity due to robust irreversible network breakdown.^48^ In addition to conductive network breakdown, irreversible microscopic fractures arise in the matrix at large strains, which further inhibit the transport efficiency. These fractures are also observed from the SEM images shown in Figure 2c. This typical trade-off between flexibility and sensing efficiency pertains to PNC systems. With a further increase in strain, the degree of increase in resistance steepens (regions: GF_2_ in PUD20 and GF_3_ in PUD15), resulting from the intensification of network breakdown. At such higher strains, the prepared films displayed GF values of 175.7 for PUD15 and 601.5 for PUD20, respectively. It is to be noted that the PUD15 film has shown three regimes of linearity with incremental GF upon strain. This characteristic response in elastomeric films is exclusively governed by their mechanical properties. Table 2 compares the working strain GF of similar PNC systems with our results.

**Table 2:** Performance Comparison of the Current Work with Similar TPU-Based Carbon Nanocomposites, which are Prepared through Solution-Mediated Filler Dispersion^a^

Composite material	Gauge factor (GF_1_)	Working strain range (in %)	Ref
TPU/MWCNT	22	up to 45	^49^
TPU/CNT	11.3	20–250	^50^
TPU/Graphene nanoplatelet (electrospun)	4.6	up to 150	^51^
TPU/CNT and graphene bifiller	35.7	-	^52^
TPU/3D hybrid CNT/FLG	123	up to 8	This work

a GF mentioned in the table corresponds to the working strain regimes.

From the aforementioned studies on unidirectional strain, an optimal balance between flexibility and broad working (linear) range has been observed for the PUD15 composite, while an elevated small strain sensitivity has resulted in PUD20. Hence, the PUD15 composite was chosen to examine the cyclic stability under multiple stretch–compression cycles. Figure 6a shows the durability analysis conducted for 1000 cycles of loading–unloading at a strain rate of 1.5 mm/s, with a time delay of 1 s between the steps. It has been observed that the relative variation in resistance is higher in the initial cycles and normalizes later due to the reorientation of conductive networks under repeated loading–unloading. Figure 6b shows the highly stable strain response obtained for smaller strain cycles between 0% and 6.6% strain. The results indicate a very small distortion in small strain limits (linear regime) and show pronounced “shoulder peaks”.^53^ In Figure 6c, an enlarged section of Figure 6b is plotted simultaneously with its corresponding applied stress to validate the consistent response. From the said cyclic measurements, good response and recovery times are observed, with their average values being ∼570 and ∼860 ms, respectively. One could observe a decrease in resistance during the delay time (indicated with blue lines in Figure 6b), which specifies the recovery of conductive networks. Such recovery is attributed to the elastic nature of the matrix, which tends to revert to its original shape after deformation, thereby reorienting the filler structures to partially recover the disrupted networks.

**Figure 6:**
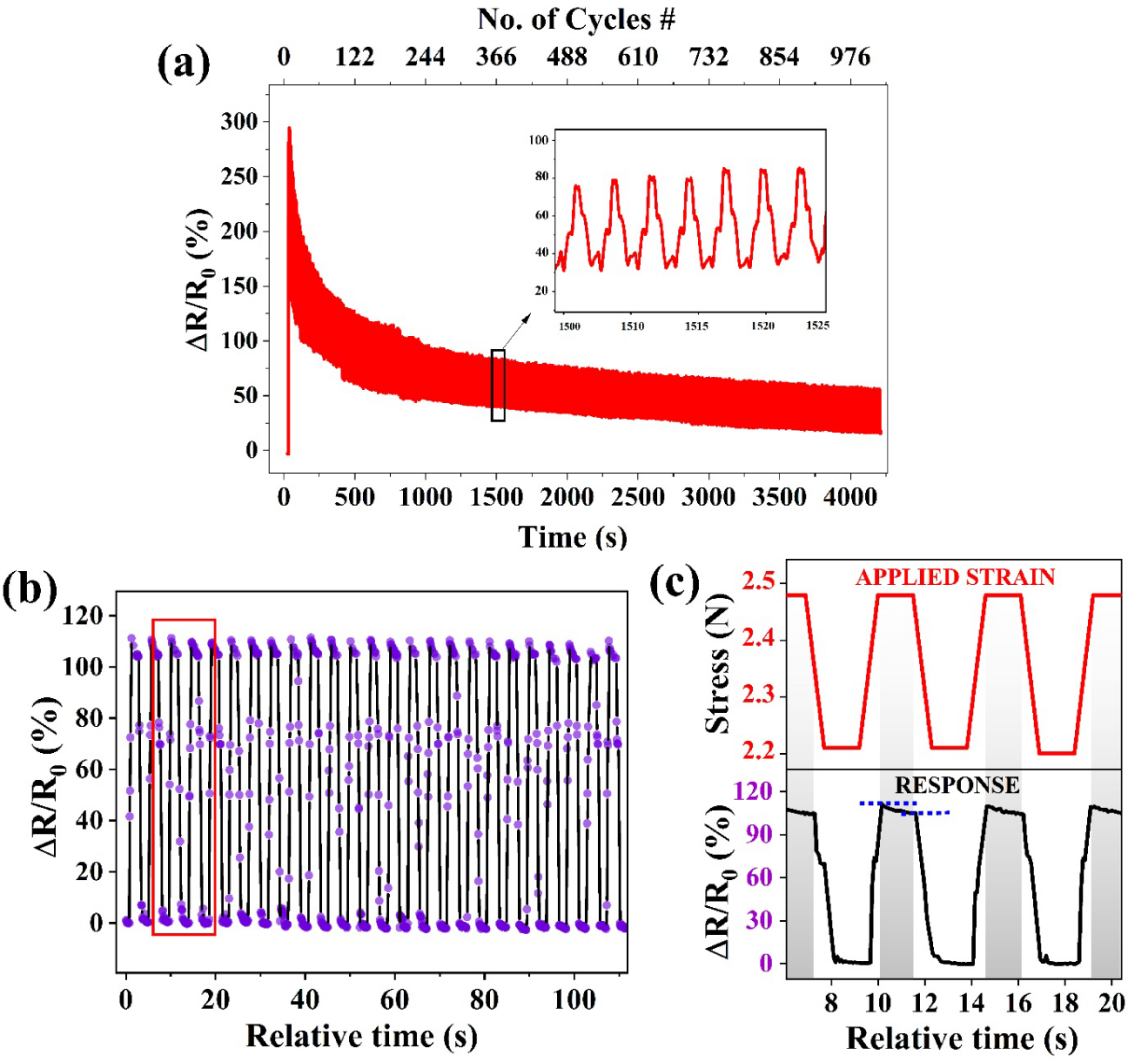
(a) Durability test conducted for 1000 cycles in PUD15 films at 12% strain. (b) Small strain-sensing behavior of the PUD15 composite between 0% and 6.6% strain, showing its sheer stability within the linear regime. (c) Simultaneous plots of applied stress and its corresponding electrical response for a representative enlarged section of (b), in which blue lines indicate the recovery of conductivity during the delay time.

## Conclusions

The functionally rich TPU elastomer, incorporated with 3D hybrid fillers, was comprehensively studied in terms of its structural, mechanical, and strain-sensing behaviors. The preliminary characterization of the solution-cast, thin, flexible composites via WAXD and Raman spectroscopy confirmed a superior reinforcement of 3D fillers within TPU matrices. Similarly, the pronounced interfacial interaction was also confirmed through mechanical analysis, which revealed a surge in storage modulus by 4.6 times that of the neat elastomer, attributed to the TPU-3D synergy. These observations further validated TPU as a promising host for multidimensional carbon nanofillers. Through deliberate structural analyses, it was shown that 3D fillers are confined in a polydisperse fashion within a polar TPU matrix. Moreover, the fitted USAXS data interpreted an incremental aggregate dimension with concentration, which possessed branched and highly irregular filler conformations. This structural aspect is considered highly favorable for the formation of conductive transport networks within the composites.

The prepared elastomers displayed excellent strain-sensing behavior in both unidirectional and cyclic strain. Herein, a 4.8-fold increase in the linear (low-strain) GF was observed upon increasing 3D loading from 15 to 20 wt %. However, a comparable surge in mechanical modulus (4.6 times) is also observed for the same increment in concentration, which has significantly limited the working (linear) strain range for the 20 wt % composite. A maximum GF of 601 was achieved for strain above 8% (high strain) in 20 wt % 3D loading. This high GF in the working strain range, along with the short response and recovery times, corroborates that the TPU/3D composites are highly sensitive and are exclusively applicable for small-strain monitoring.

We observe that this significant amount of nanofiller dispersion within the TPU matrix has enhanced the mechanical integrity and sensing stability under cyclic deformation. Nevertheless, due to the inherent trade-off between mechanical rigidity and elastic conformability in PNCs, simply adding nanofillers is not enough to meet the requirements for strain-sensing applications. Therefore, a structure-driven approach emphasizes the importance of optimizing interactions to achieve a suitable design for wearable strain-sensing application systems. Additionally, the pertaining scalability issues in the Doctor blade method need to be addressed in the future for large-scale production of conductive sensors.
